# Drug-induced eosinophilic pneumonia: a real-world data mining study of the FAERS and Vigibase database reveals potential safety signals and risk patterns

**DOI:** 10.3389/fmed.2026.1788843

**Published:** 2026-04-22

**Authors:** Jing Hu, Yao Sun, Cong Cheng, Sisi Wang, Xiangrong Zuo, Yun Liu

**Affiliations:** 1 Department of Pharmacy, The First Affiliated Hospital of Nanjing Medical University, Nanjing, Jiangsu, China; 2 Department of Pharmacy, Children’s Hospital of Nanjing Medical University, Nanjing, Jiangsu, China; 3 Department of Pharmacy, The Affiliated Lianyungang Hospital of Xuzhou Medical University, Lianyungang, Jiangsu, China; 4 Department of Critical Care Medicine, The First Affiliated Hospital of Nanjing Medical University, Nanjing, Jiangsu, China

**Keywords:** disproportionality analysis, eosinophilic pneumonia, FDA adverse event reporting system, pharmacovigilance, retrospective study

## Abstract

**Background:**

Eosinophilic pneumonia (EP) is an uncommon, idiopathic interstitial lung disease distinguished by the atypical accumulation of eosinophils within the pulmonary parenchyma and airways. The condition often presents insidiously and is frequently overlooked or misdiagnosed; pharmacological agents are among the acknowledged precipitants of this disorder.

**Methods:**

This is a retrospective pharmacovigilance investigation. We utilized the FAERS and Vigibase databases to detect adverse reactions associated with drug-related EP.

**Results:**

We identified a total of 15,374 cases of drug-induced EP in FAERS. The incidence was highest, at 24.1%, among individuals aged 45 to 64 years, with 35.4% of the affected patients requiring hospitalization. In terms of the numerical composition of PTs, pneumonitis was the most predominant PT. Although the proportion of PTs varied for each drug and pneumonitis remained the most common, antibacterials exhibited a higher prevalence of “eosinophilic pneumonia” and “pulmonary eosinophilia.” Notable observations include significant signal variations between the two databases for certain drugs, yet all positive signal drugs identified by FAERS can be confirmed by Vigibase. Initial screening identified 302 suspect drugs; following disproportionality filtering, univariate analysis, and lasso shrinkage, 56 agents were retained. Nivolumab was the most frequently reported drug (1,377 reports), followed by pembrolizumab (1,070 reports) and daptomycin (758 reports), with daptomycin exhibiting the most significant statistical signal in FAERS. Time-to-onset analysis indicated that EP typically manifested early. Multivariable modeling identified higher body weight, advancing age, and polypharmacy as associated factors. The drugs most strongly associated with EP were daptomycin (OR 12.50, 95% CI 9.40–16.75), durvalumab (OR 5.17, 95% CI 3.74–7.14), fam-trastuzumab deruxtecan (OR 4.86, 95% CI 3.32–7.04), idelalisib (OR 4.74, 95% CI 3.13–7.07), and osimertinib (OR 3.21, 95% CI 2.11–4.79).

**Conclusion:**

The early discontinuation of the offending drug, timely initiation of corticosteroid therapy, and multidisciplinary collaboration are fundamental to achieving improved outcomes in cases of drug-induced eosinophilic pneumonia. This study offers substantial real-world evidence to facilitate the early identification and optimal management of eosinophilic pneumonia.

## 1 Introduction

Acute eosinophilic pneumonia (AEP) is a rare respiratory disorder characterized by acute onset and variable severity that can range from mild illness to life-threatening acute respiratory distress syndrome (ARDS), potentially resulting in mortality (1). Eosinophils (EOS) induce tissue damage of EP primarily through two mechanisms: (1) oxidative stress mediated by eosinophil peroxidase (EPO) and major basic protein (MBP), which disrupts the extracellular matrix; and (2) the action of granule proteins, such as eosinophil cationic protein (ECP) or through antibody-dependent cell-mediated cytotoxicity (ADCC). Furthermore, EOS infiltration directly promotes fibrosis releasing of transforming growth factor-*β* (TGF-β), interleukin-4 (IL-4), and interleukin-13 (IL-13), and indirectly by inducing the expression of profibrotic mediators in epithelial cells via EPO/MBP (2–4). These complex mechanisms render the clinical assessment and diagnostic decision-making for EOS-mediated diseases particularly challenging. AEP is generally uncommon, with limited epidemiological data available. Studies in military cohorts indicate an incidence rate of 9.1–11.0 cases per 100,000 person-years (9.1 in the United States and 11.0 in Korea). Although AEP can be idiopathic, recognized triggers include smoking, various inhalational exposures, medications, and infections. Clinical case series indicate that 31% of cases are smoking-related, 17% are drug-induced, and the remainder are idiopathic (5–7). A wide range of medications, particularly antibiotics, nonsteroidal anti-inflammatory drugs (NSAIDs), and selective serotonin reuptake inhibitors (SSRIs), have been implicated in AEP, with most evidence originating from case reports (8–10). A prior analysis of the FDA Adverse Event Reporting System (FAERS) data, which concentrated on anti-methicillin-resistant Staphylococcus aureu (anti-MRSA) agents, identified daptomycin as a factor that increases the risk of EP (11). However, this investigation did not comprehensively assess the associations between all drugs reported in FAERS and Vigibase. Leveraging the largest global pharmacovigilance database, this study systematically examines the reporting patterns of eosinophilic pneumonia (EP) associated with various drugs and identifies potential clinical associated factors.

## 2 Materials and methods

### 2.1 Data source and collection

This study was conducted and reported in strict accordance with the READUS-PV (Reporting of Spontaneous Adverse Drug Reaction Studies in Pharmacovigilance) guidelines (12). This study utilized data from the FAERS covering the period from the first quarter of 2014 to the first quarter of 2025. The data includes seven structured datasets: DEMO (demographic and administrative information), REAC (MedDRA-coded adverse events), DRUG (medication details), OUTC (clinical outcomes), RPSR (report sources), THER (dates of drug therapy), and INDI (MedDRA-coded indications). According to FDA deduplication guidelines, records with duplicate Case IDs were merged by keeping those with the most recent FDA receipt date (fda_dt). When both the Case ID and fda_dt were the same, the entry with the largest Primary ID was retained. A secondary manual deduplication process was subsequently carried out to handle any remaining duplicate reports based on identical patient demographics (age, sex) and event dates. Additionally, reports missing key details like age, sex, or medication/adverse event dates were excluded from the main analysis. Adverse events were characterized using the Medical Dictionary for Regulatory Activities (MedDRA version 25.0). Cases of EP were identified using the Standardized MedDRA Query (SMQ 20000159 “Eosinophilic pneumonia”), which encompasses 15 Preferred Terms, such as eosinophilic pneumonia (PT 10014962), its acute and chronic variants (PT 10052832/10052833), Löffler’s syndrome (PT 10024794), Pneumonitis(PT 10035742), pulmonary eosinophilia (PT 10037382), hypereosinophilic syndrome (PT 10048643), eosinophilic granulomatosis with polyangiitis (PT 10078117), and related manifestations, including eosinophilic pleural effusion (PT 10080148). Only reports designating drugs as primary suspects were retained. Vigibase data were utilized to validate the reliability of FAERS data. Data extraction from Vigibase involved SMQ 20000159 ‘Eosinophilic pneumonia’, with relevant Preferred Terms used for computational verification.

### 2.2 Determination of the drug list

In formulating our signal detection strategy, we drew upon the methodological framework outlined by Duke et al., which encapsulates essential detection algorithms and critical considerations pertinent to studies utilizing spontaneous reporting systems (13). A two-by-two contingency table was constructed to evaluate the association between EP and suspected drugs through disproportionality analysis. Drugs with a therapeutic effect on EP were excluded from the analysis. This study employed a multi-algorithmic approach, integrating frequentist methods—Reporting Odds Ratio (ROR) and Proportional Reporting Ratio (PRR)—with the Bayesian Information Component (IC) method. This strategy aims to balance sensitivity and specificity: ROR and PRR are sensitive for early signal detection, while the Bayesian IC, using a “shrinkage” mechanism, provides stable estimates for rare events, minimizing false positives. A positive signal was determined if any of these methods indicated significance. Specifically, a PRR signal was defined by a report count of ≥3, PRR ≥ 2, and χ^2^ ≥ 4, while a positive ROR signal was identified when the lower limit of the two-sided 95% CI was >1. The IC method detected signals using the IC025 metric, where an IC025 value exceeding 0 indicated a potential association between the drug and the event (13). The *p*-adjusted value, obtained from Fisher’s exact test followed by Bonferroni correction, was used for further analysis. Subsequently, a volcano plot was constructed to visually characterize the relationship between signal strength and statistical significance, utilizing –log(p-adjust) as the abscissa and log(ROR) as the ordinate.

### 2.3 LASSO and multivariable logistic regression

Pharmaceutical agents were evaluated based on the event_date and start_date to determine the duration until the onset of adverse events. Prior to the TTO analysis, data were screened for temporal completeness. Reports with missing, incomplete, or logically aberrant date information (e.g., event dates preceding start dates resulting in negative TTO values) were excluded from the survival and TTO distributions analyses; however, these cases were retained for the primary disproportionality signal detection. A Weibull parametric survival model was subsequently utilized to assess the temporal risk relationship between drug exposure and the development of eosinophilic pneumonia. Kruskal–Wallis tests were used to compare TTO distributions across ADE groups, and Kaplan–Meier curves were generated to visualize the cumulative incidence of ADEs over time. Drugs identified as significant—characterized by a lower limit of the 95% confidence interval for the ROR exceeding 1, more than three reported cases, and an adjusted *p*-value of less than 0.01 in univariate analysis—were incorporated into the least absolute shrinkage and selection operator (LASSO) regression for variable selection. Subsequently, multivariable logistic regression was performed to explore potential correlates associated with eosinophilic pneumonia reports.

### 2.4 Statistical analysis

Descriptive statistics were employed to summarize and present the clinical characteristics of patients reported with eosinophilic pneumonia. Drugs potentially associated with eosinophilic pneumonia were initially screened using measures such as the reporting odds ratio (ROR). Candidate variables were then refined through LASSO regression, with the tuning parameter *λ* selected by 10-fold cross-validation to minimize the mean squared error. Variables with non-zero coefficients were subsequently included in a multivariable logistic regression model. All analyses were conducted in R (version 4.2.1).

## 3 Results

### 3.1 Demographic characteristics for drug induced EP

Data processing revealed a total of 15,374 reported cases of eosinophilic pneumonia (EP) from Q1 2014 to Q1 2025 (Table 1). Among these EP cases, the reporting rate for males was slightly higher than that for females (42.6% vs. 41.5%), and the 45–64-year age group had the highest incidence at 24.1%. The majority of cases originated from the United States, Canada, Japan, and other countries. Hospitalization was the most frequently reported outcome, accounting for 35.4% of all submitted records. A total of 302 drugs were associated with EP cases.

**Table 1:** Clinical and demographic characteristics of drug-induced EP.

Characteristic	EP	ALL
Number of reports	15,374	1,518,139
Gender, *n* (%)		
Male	6,382 (41.5%)	539,224 (35.5%)
Female	6,554 (42.6%)	747,870 (49.3%)
Unknown	2,438 (15.9%)	231,045 (15.2%)
Weight, kg, *n* (%)		
<50 kg	391 (2.5%)	41,047 (2.7%)
50–70 kg	1,642 (10.7%)	141,013 (9.3%)
70–90 kg	1,508 (9.8%)	112,390 (7.4%)
90–110 kg	560 (3.6%)	42,954 (2.8%)
>110 kg	221 (1.4%)	20,188 (1.3%)
Unknown	11,052 (71.9%)	1,160,547 (76.4%)
Age, years, *n* (%)		
<18	296 (1.9%)	59,620 (3.9%)
18–44	994 (6.5%)	130,152 (8.6%)
45–64	3,706 (24.1%)	347,153 (22.9%)
65–74	3,358 (21.8%)	245,979 (16.2%)
>75	2022 (13.2%)	164,157 (10.8%)
Unknown	4,998 (32.5%)	571,078 (37.6%)
Reporting year, *n* (%)		
2014–2020	4,071 (26.5%)	440,993 (29.0%)
2021	744 (4.8%)	66,982 (4.4%)
2022	688 (4.5%)	74,343 (4.9%)
2023	603 (3.9%)	67,689 (4.5%)
2024	380 (2.5%)	43,005 (2.8%)
Unknown	8,888 (57.8%)	821,280 (54.1%)
Country, *n* (%)		
America	5,989 (39.0%)	627,006 (41.3%)
Japan	1,232 (8.0%)	122,440 (8.1%)
Canada	1,183 (7.7%)	127,323 (8.4%)
Germany	1,058 (6.9%)	68,252 (4.5%)
United Kingdom	825 (5.4%)	69,956 (4.6%)
Spain	544 (3.5%)	28,712 (1.9%)
Italy	542 (3.5%)	53,557 (3.5%)
Frich	411 (2.7%)	94,302 (6.2%)
China	390 (2.5%)	50,459 (3.3%)
Other	3,200 (20.8%)	276,132 (18.2%)
Serious adverse event, *n* (%)		
Hospitalization	5,448 (35.4%)	366,698 (24.2%)
Other	4,646 (30.2%)	529,642 (34.9%)
Death	3,332 (21.7%)	244,736 (16.1%)
Life-threatening	1,226 (8.0%)	60,919 (4.0%)
Disability	646 (4.2%)	297,630 (19.6%)
Discontinued	66 (0.4%)	17,074 (1.1%)
Required intervention	10 (0.1%)	1,440 (0.1%)

### 3.2 Disproportionality and univariate analysis

**Figure 1:**
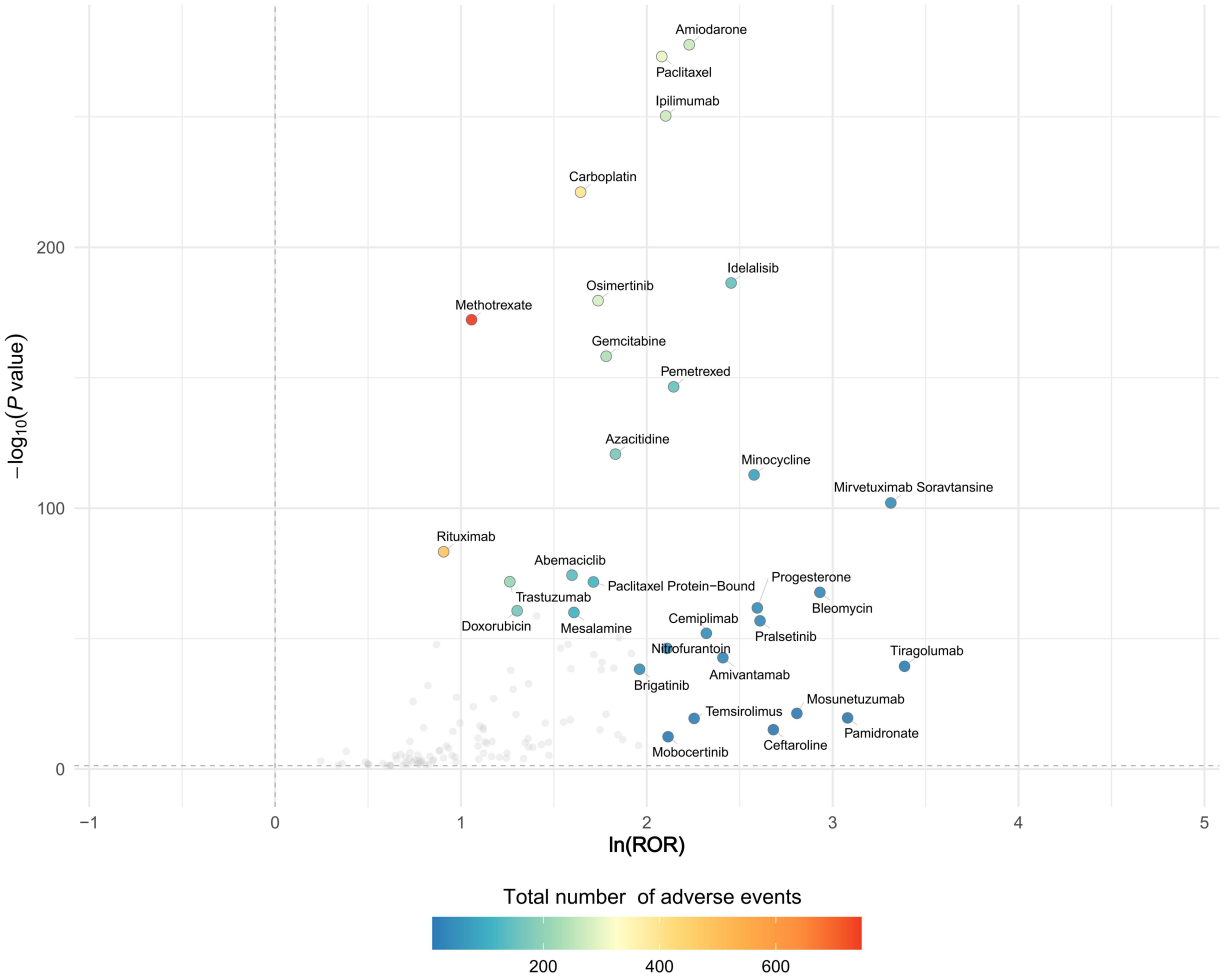
The volcano plot of adjusted *p*-value and ROR among drug-induced EP. The horizontal coordinate shows the log_2_ROR value and the vertical coordinate indicates the adjusted *p*-value after -log_10_ conversion. Adverse reaction report quantities are marked with different colors based on their magnitude. The *p*-value is adjusted with false discovery rate (FDR) method.

**Figure 2:**
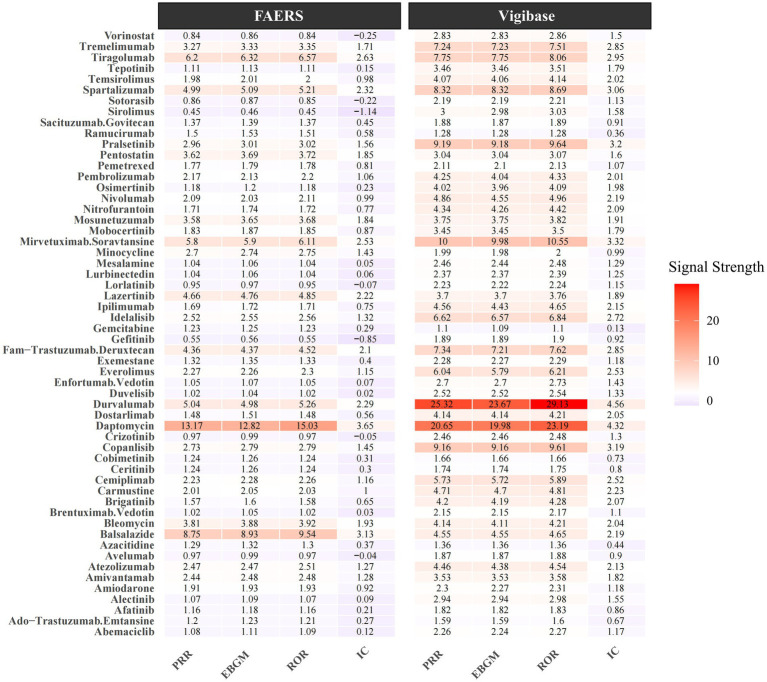
This figure presents a comparative analysis of drug safety signals between the FAERS and Vigibase pharmacovigilance databases. The chart displays various pharmaceutical compounds in the leftmost column, with their corresponding signal strength metrics organized in a tabular format with color-coded heatmap visualization.

**Figure 3:**
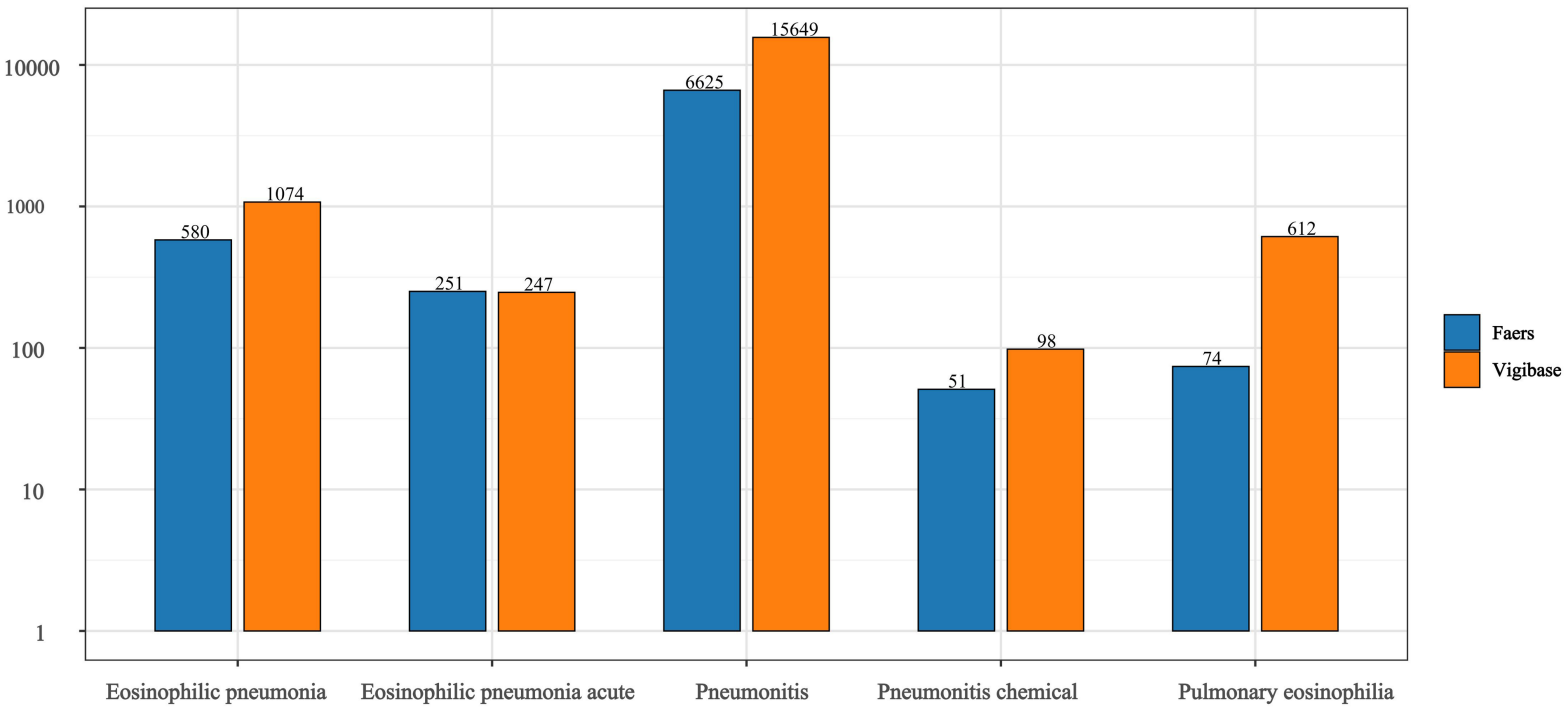
The reporting frequencies of five pulmonary conditions were compared between the FAERS and Vigibase databases. It shows that reports in Vigibase (orange) are considerably higher than in FAERS (blue) for most conditions, with “Pneumonitis” being the most reported.

**Figure 4:**
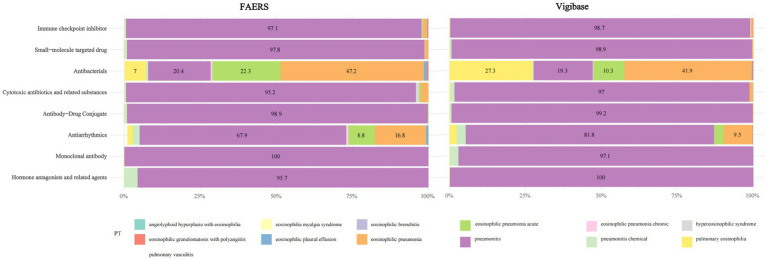
This horizontal bar chart compares the proportion of eosinophil-related pulmonary adverse reactions (PT) across drug classes between the FAERS and Vigibase databases. Each stacked bar represents a drug category, showing the distribution of specific reactions (e.g., eosinophilic pneumonia, pneumonitis). Key differences are evident; for example, monoclonal antibodies are linked solely to “pneumonitis” (100%) in FAERS, while Vigibase reports a more varied profile for antibacterials.

The volcano plot of adjusted *p*-value versus ROR, overlaid with adverse-event counts, indicates that drugs with the smallest univariate *p*-values are amiodarone, paclitaxel, and ipilimumab (Figure 1). After excluding agents with fewer than three reports, a 95% CI lower limit of ROR < 1, and a *p*-value > 0.05 in univariate analysis, 182 drugs remained. In the FAERS database, Nivolumab ranked first with 1,377 reports, followed by pembrolizumab (1,070 reports) and daptomycin (758 reports). Daptomycin showed the strongest signal, with an ROR (95% CI) of 15.03 (13.90–16.25); durvalumab ranked second at 5.26 (4.84–5.72) in FAERS. Additional details are provided in Table 2. Vigibase data showed consistent reporting patterns with FAERS, with nivolumab being the most frequently reported drug, followed by durvalumab. A notable difference was observed in signal strength, where durvalumab exhibited the strongest signals in Vigibase. Nevertheless, all positive signal drugs identified in FAERS were corroborated by Vigibase, as shown in Figure 2. Figure 3 demonstrated significantly higher reporting frequencies in Vigibase compared to FAERS across most pulmonary conditions, especially for pneumonitis and pulmonary eosinophilia. The side-by-side bar charts revealed consistent patterns of pulmonary adverse event distribution across drug classes between the two databases, while highlighting proportional differences in specific reaction types, as shown in Figure 4. Key differences were evident; for example, monoclonal antibodies are linked solely to “pneumonitis” (100%) in FAERS, while Vigibase reports a more varied profile for antibacterials.

**Table 2:** Disproportionality analysis of drug induced EP in the FAERS and Vigibase database.

Drugs	FAERS	PRR (χ2)	EBGM (EBGM05)	ROR (95%CI)	IC (IC025)	Vigibase	PRR (χ2)	EBGM (EBGM05)	ROR (95%CI)	IC (IC025)
Nivolumab	1,377	2.09 (720.14)	2.03 (1.92)	2.11 (2.00–2.23)	0.99 (0.94)	2,731	4.86 (7742.12)	4.55 (4.37)	4.96 (4.77–5.16)	2.19 (2.10)
Pembrolizumab	1,070	2.17 (635.66)	2.13 (2.00)	2.20 (2.06–2.34)	1.06 (1.00)	2,213	4.25 (5176.18)	4.04 (3.87)	4.33 (4.14–4.52)	2.01 (1.93)
Daptomycin	758	13.17 (8187.51)	12.82 (11.86)	15.03 (13.90–16.25)	3.65 (3.38)	1,174	20.65 (21322.12)	19.98 (18.78)	23.19 (21.80–24.66)	4.32 (4.06)
Everolimus	700	2.27 (479.73)	2.26 (2.09)	2.30 (2.13–2.48)	1.15 (1.06)	1722	6.04 (6920.28)	5.79 (5.51)	6.21 (5.92–6.53)	2.53 (2.41)
Durvalumab	610	5.04 (1917.42)	4.98 (4.58)	5.26 (4.84–5.72)	2.29 (2.10)	2,327	25.32 (50954.69)	23.67 (22.62)	29.13 (27.84–30.47)	4.56 (4.36)
Atezolizumab	563	2.47 (480.51)	2.47 (2.27)	2.51 (2.31–2.73)	1.27 (1.17)	766	4.46 (2018.76)	4.38 (4.07)	4.54 (4.23–4.89)	2.13 (1.98)
Fam-trastuzumab deruxtecan	341	4.36 (872.05)	4.37 (3.92)	4.52 (4.05–5.04)	2.10 (1.88)	706	7.34 (3810.17)	7.21 (6.68)	7.62 (7.06–8.22)	2.85 (2.64)
Osimertinib	282	1.18 (7.30)	1.20 (1.06)	1.18 (1.05–1.33)	0.23 (0.20)	692	4.02 (1545.76)	3.96 (3.67)	4.09 (3.79–4.41)	1.98 (1.84)
Ipilimumab	271	1.69 (76.59)	1.72 (1.52)	1.71 (1.51–1.93)	0.75 (0.66)	1,179	4.56 (3179.20)	4.43 (4.18)	4.65 (4.38–4.93)	2.15 (2.03)
Amiodarone	267	1.91 (115.21)	1.93 (1.71)	1.93 (1.71–2.18)	0.92 (0.82)	730	2.30 (525.32)	2.27 (2.11)	2.31 (2.15–2.49)	1.18 (1.10)
Gemcitabine	236	1.23 (10.00)	1.25 (1.10)	1.23 (1.08–1.40)	0.29 (0.26)	600	1.10 (4.98)	1.09 (1.01)	1.10 (1.01–1.19)	0.13 (0.12)
Azacitidine	170	1.29 (11.37)	1.32 (1.13)	1.30 (1.12–1.51)	0.37 (0.32)	163	1.36 (15.60)	1.36 (1.16)	1.36 (1.17–1.59)	0.44 (0.38)
Pemetrexed	151	1.77 (50.18)	1.79 (1.53)	1.78 (1.51–2.09)	0.81 (0.69)	432	2.11 (252.13)	2.10 (1.91)	2.13 (1.93–2.34)	1.07 (0.97)
Idelalisib	150	2.52 (137.28)	2.55 (2.17)	2.56 (2.17–3.01)	1.32 (1.12)	254	6.62 (1208.89)	6.57 (5.80)	6.84 (6.03–7.75)	2.72 (2.40)
Minocycline	81	2.70 (87.04)	2.74 (2.20)	2.75 (2.20–3.43)	1.43 (1.14)	196	1.99 (96.16)	1.98 (1.72)	2.00 (1.74–2.30)	0.99 (0.86)
Exemestane	69	1.32 (5.50)	1.35 (1.06)	1.33 (1.05–1.69)	0.40 (0.32)	174	2.28 (124.64)	2.27 (1.95)	2.29 (1.97–2.66)	1.18 (1.02)
Ramucirumab	57	1.50 (9.61)	1.53 (1.18)	1.51 (1.16–1.96)	0.58 (0.45)	84	1.28 (5.25)	1.28 (1.03)	1.28 (1.04–1.59)	0.36 (0.29)
Sacituzumab govitecan	55	1.37 (5.49)	1.39 (1.07)	1.37 (1.05–1.79)	0.45 (0.35)	61	1.88 (25.04)	1.87 (1.46)	1.89 (1.46–2.43)	0.91 (0.70)
Nitrofurantoin	49	1.71 (14.53)	1.74 (1.31)	1.72 (1.30–2.29)	0.77 (0.58)	796	4.34 (2011.12)	4.26 (3.97)	4.42 (4.12–4.75)	2.09 (1.95)
Brigatinib	47	1.57 (9.78)	1.60 (1.20)	1.58 (1.18–2.11)	0.65 (0.49)	74	4.20 (180.83)	4.19 (3.33)	4.28 (3.39–5.39)	2.07 (1.64)
Cemiplimab	46	2.23 (31.57)	2.28 (1.70)	2.26 (1.69–3.03)	1.16 (0.86)	100	5.73 (391.67)	5.72 (4.68)	5.89 (4.83–7.19)	2.52 (2.06)
Mirvetuximab soravtansine	46	5.80 (183.92)	5.90 (4.38)	6.11 (4.53–8.23)	2.53 (1.88)	60	10.00 (487.93)	9.98 (7.69)	10.55 (8.12–13.69)	3.32 (2.56)
Pralsetinib	40	2.96 (52.31)	3.01 (2.20)	3.02 (2.21–4.14)	1.56 (1.14)	49	9.19 (359.08)	9.18 (6.88)	9.64 (7.23–12.86)	3.20 (2.40)
Bleomycin	38	3.81 (79.23)	3.88 (2.80)	3.92 (2.83–5.43)	1.93 (1.39)	277	4.14 (657.70)	4.11 (3.65)	4.21 (3.74–4.75)	2.04 (1.81)
Amivantamab	35	2.44 (29.94)	2.48 (1.78)	2.48 (1.77–3.47)	1.28 (0.92)	56	3.53 (102.10)	3.53 (2.71)	3.58 (2.75–4.67)	1.82 (1.40)
Temsirolimus	18	1.98 (8.75)	2.01 (1.26)	2.00 (1.25–3.18)	0.98 (0.62)	90	4.07 (209.09)	4.06 (3.30)	4.14 (3.36–5.11)	2.02 (1.64)
Tiragolumab	17	6.20 (74.88)	6.32 (3.87)	6.57 (4.01–10.74)	2.63 (1.61)	9	7.75 (53.18)	7.75 (3.97)	8.06 (4.13–15.73)	2.95 (1.51)
Mobocertinib	13	1.83 (4.95)	1.87 (1.08)	1.85 (1.07–3.20)	0.87 (0.50)	10	3.45 (17.53)	3.45 (1.85)	3.50 (1.87–6.55)	1.79 (0.96)
Mosunetuzumab	13	3.58 (24.45)	3.65 (2.10)	3.68 (2.12–6.41)	1.84 (1.06)	11	3.75 (22.35)	3.75 (2.07)	3.82 (2.10–6.93)	1.91 (1.05)

ROR: reporting odds ratio; CI: confidence interval; PRR: proportional reporting ratio; χ^2^ chi-squared; IC: information component; IC025: the lower limit of 95% CI of the IC; EBGM: empirical Bayesian geometric mean; EBGM05: the lower limit of 95% CI of EBGM.

### 3.3 The time-to-onset (TTO) analysis of drug induced EP

**Figure 5:**
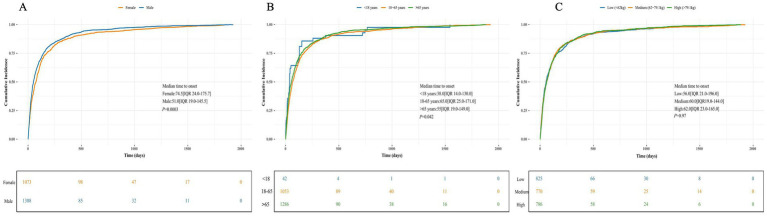
Cumulative distribution functions of time-to-onset for drug induced EP in FAERS database. The figure shows the time-to-onset distribution patterns for EP associated drug. IQR, interquartile range. **(A)** Cumulative incidence curve comparing time to onset by gender; **(B)** Stratified by age group; **(C)** By weight category.

Among the total 15,374 identified drug induced EP, 13,021 (84.7%) were excluded from the time-to-onset (TTO) analyses due to missing or invalid start_date or event_date information. Consequently, the TTO evaluation was conducted on the remaining 2,353 cases with complete temporal data. The time-to-onset (TTO) analysis based on the Weibull distribution indicated that eosinophilic pneumonia generally followed an “early-failure” pattern (Table 3). At the drug level, nivolumab (*n* = 298; median onset: 70 days; shape parameter: 0.77), atezolizumab (*n* = 171; median onset: 45 days; shape parameter: 0.77), daptomycin (*n* = 159; median onset: 19 days; shape parameter: 0.93), pembrolizumab (*n* = 138; median onset: 69 days; shape parameter: 0.76), and ipilimumab (*n* = 99; median onset: 71 days; shape parameter: 0.77) all demonstrated early-failure characteristics. In contrast, durvalumab (*n* = 77; shape parameter: 1.14) and idelalisib (*n* = 25; shape parameter: 1.23) showed a “wear-out” failure profile. Subgroup time-trend analyses revealed that both sexes followed an early-failure pattern, yet a significant difference was noted between males and females (Figure 5). The two Kaplan–Meier curves became parallel after approximately 150 days, suggesting that the sex disparity is mainly limited to the first 5 months after drug exposure. Males experience adverse events significantly earlier (median time: 51.0 days) than females (median time: 74.5 days). The scale parameters suggest that the youngest group (<18 years) has the shortest characteristic time to event (38.0 days), while the 18–65 years group has the longest (65.0 days). However, the confidence intervals for scale parameters overlap somewhat, implying some uncertainty in these estimates. All three age curves reached a plateau after 200 days, indicating that advanced age delays the occurrence of EP. In contrast, subgroup analyses based on body weight revealed no significant differences in the time trend.

**Table 3:** Time-to-onset analysis of drug-induced EP using Weibull distribution test in the FAERS database

Drug	**Time-to-onset(days)**			**Weibull distribution**				**Failure type**
				**Scale parameter**		**Shape parameter**		
	**N**	**Median(IQR)**	**Min-Max**	**α**	**95%CI**	**β**	**95%CI**	
Nivolumab	298	70 (24–224)	1–1,344	129.69	110.11–129.97	0.77	0.71–0.78	Early failure
Atezolizumab	171	45 (18–131)	1–1,252	90.23	74.00–90.77	0.77	0.70–0.78	Early failure
Daptomycin	159	19 (10–24)	1–749	23.47	19.65–23.57	0.93	0.83–0.94	Early failure
Pembrolizumab	138	69 (24–176)	1–1,173	125.36	97.75–125.58	0.76	0.68–0.77	Early failure
Ipilimumab	99	71 (26–148)	2–1,485	125.24	96.76–124.28	0.77	0.67–0.78	Early failure
Durvalumab	77	60 (28–100)	1–316	81.78	65.47–82.36	1.14	0.96–1.15	Wear-out failure
Everolimus	72	89 (27–194)	10–1,436	149.89	109.38–151.94	0.79	0.66–0.80	Early failure
Osimertinib	50	50 (18–99)	4–1,328	110.03	64.34–110.40	0.71	0.60–0.73	Early failure
Fam-trastuzumab deruxtecan	33	146 (74–198)	2–1,480	233.05	146.96–232.69	0.88	0.69–0.90	Early failure
Gemcitabine	26	51 (43–73)	3–905	91.91	55.84–92.19	0.87	0.67–0.91	Early failure
Idelalisib	25	90 (62–174)	14–445	149.56	105.06–151.62	1.23	0.95–1.27	Wear-out failure
Amiodarone	19	34 (12–212)	1–1,102	120.72	57.09–121.27	0.60	0.44–0.63	Early failure
Azacitidine	19	40 (10–102)	4–553	66.22	33.12–67.07	0.73	0.54–0.78	Early failure
Pemetrexed	18	49 (39–105)	10–820	119.10	65.23–119.76	0.84	0.63–0.89	Early failure
Ramucirumab	13	101 (52–140)	8–391	125.95	71.46–127.92	1.06	0.78–1.14	Wear-out failure
Tiragolumab	10	74 (44–218)	6–320	130.71	66.26–131.89	1.09	0.75–1.20	Wear-out failure
Sacituzumab govitecan	9	87 (20–153)	12–561	115.19	46.22–119.74	0.86	0.58–0.97	Early failure
Exemestane	8	174 (97–297)	31–373	219.50	127.41–220.40	1.56	1.05–1.77	Wear-out failure
Brigatinib	8	10(7–28)	1–128	22.70	7.55–23.69	0.73	0.50–0.82	Early failure
Nitrofurantoin	7	8(2–206)	1–529	44.53	5.33–49.42	0.43	0.27–0.49	Early failure
Mosunetuzumab	7	82(53–215)	13–649	160.74	54.73–167.68	0.86	0.55–1.00	Early failure
Amivantamab	6	632(148–1,117)	8–1,451	591.04	188.29–605.74	0.81	0.52–0.98	Early failure
Temsirolimus	6	20(15–74)	8–115	43.96	17.20–46.16	1.07	0.69–1.28	Wear-out failure
Minocycline	4	19(15–20)	3–24	17.67	9.49–18.11	2.02	1.22–2.77	Wear-out failure
Pralsetinib	4	79(56–386)	46–1,249	255.15	36.47–275.96	0.70	0.41–0.98	Early failure

CI, confidence interval; IQR, interquartile range; α, scale parameter; β, shape parameter.

### 3.4 Multivariate regression analysis of drug induced EP

**Figure 6:**
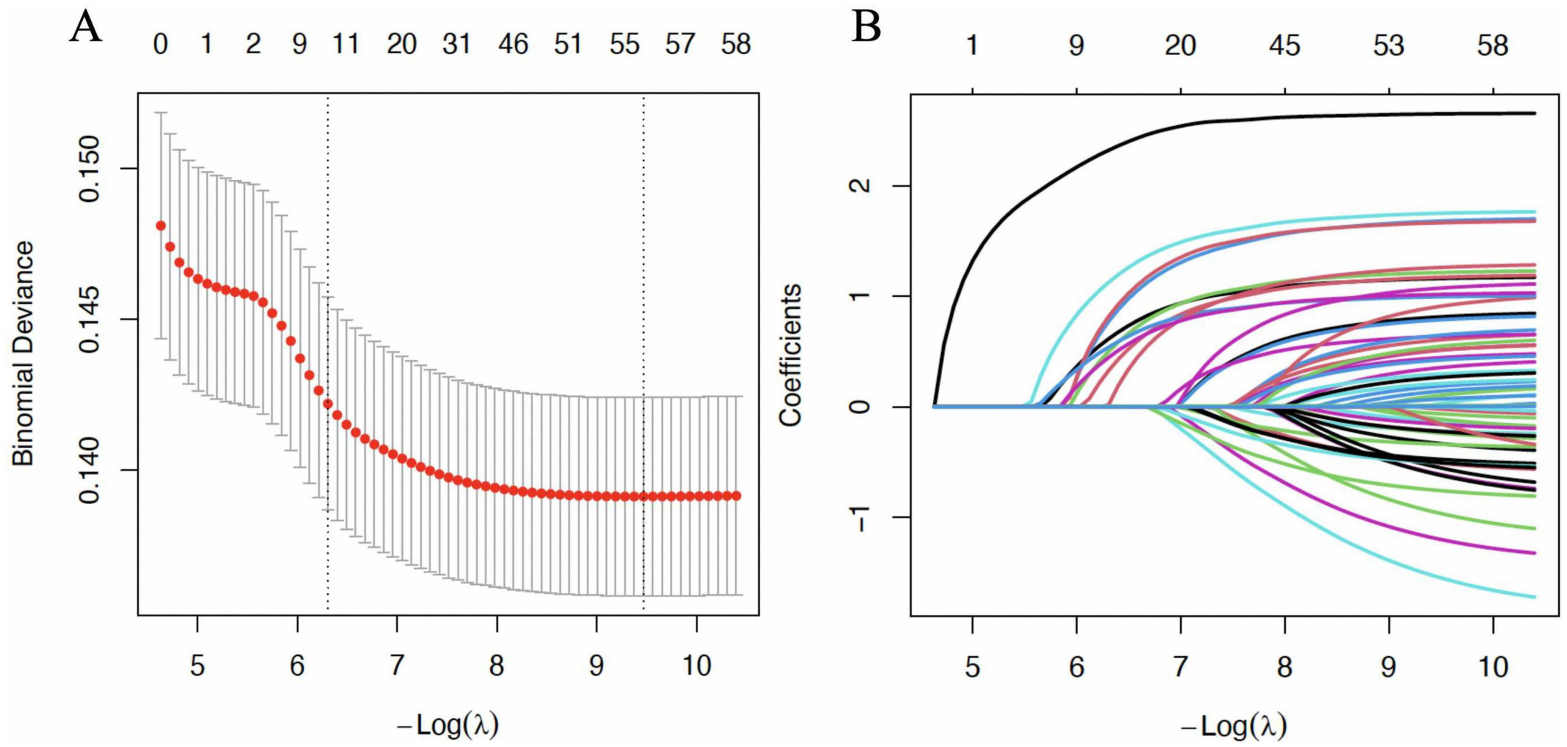
Results of the LASSO regression analysis. LASSO, least absolute shrinkage and selection operator. **(A)** The process of selection. **(B)** Average deviation and confidence interval.

**Figure 7:**
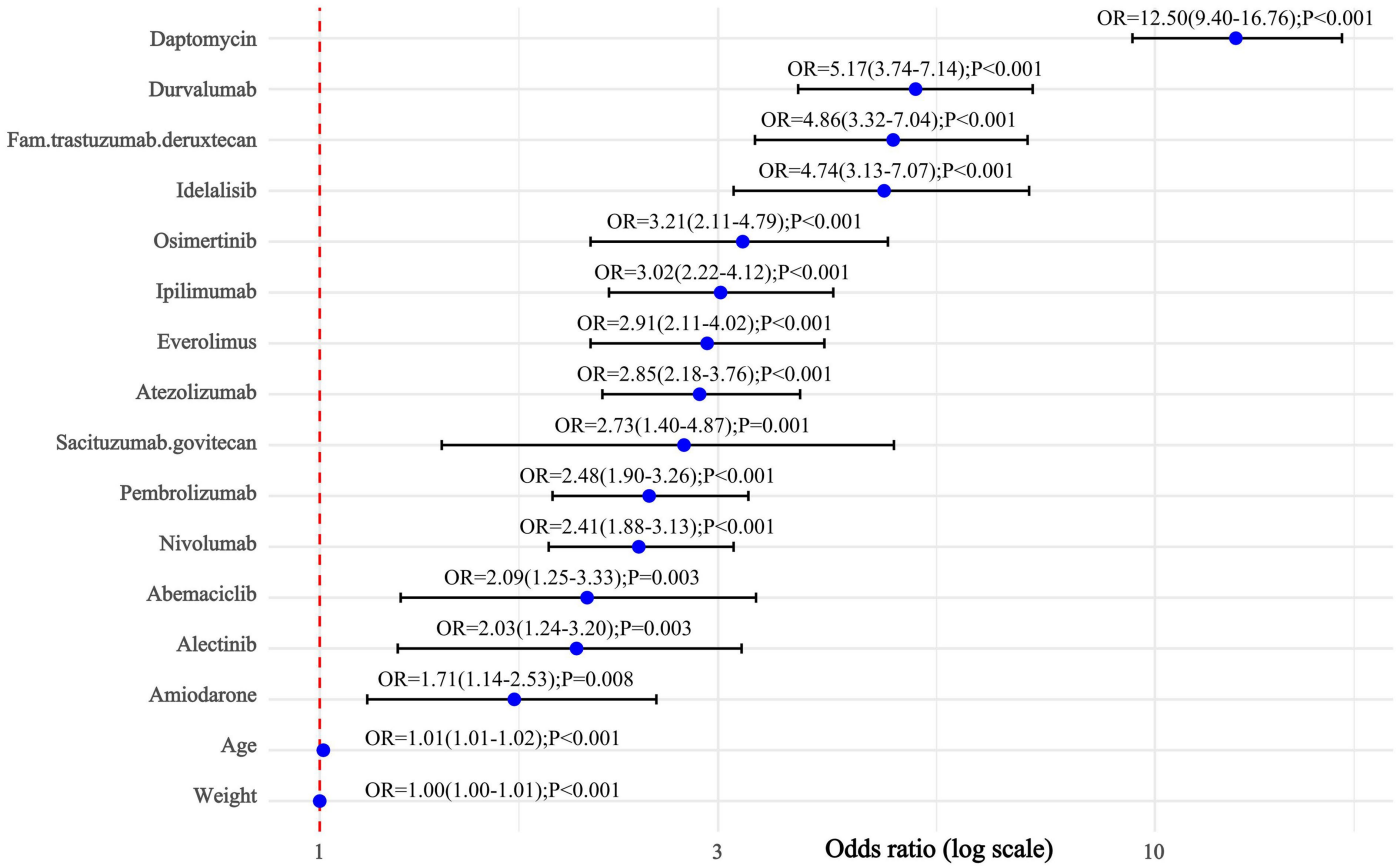
Results of the multi-factor logistic regression analysis. CI, confidence interval; OR, odds ratio; *p*-value, *p*-value after Bonferroni correction; *p*-value <0.01, statistically significant.

This multivariate regression analysis revealed that body weight, age, and multiple medications are independent factors associated with EP after LASSO regression (Figure 6). Specifically, increases in body weight and age significantly elevate the risk of the outcome, with age having a particularly pronounced effect (OR = 1.01). In the multivariate regression analysis, several medications were identified as associated factors, including amiodarone, alectinib, abemaciclib, nivolumab, pembrolizumab, sacituzumab, atezolizumab, everolimus, ipilimumab, osimertinib, and idelalisib (Figure 7). Notably, daptomycin (OR = 12.50, 95% CI = 9.40–16.75), durvalumab (OR = 5.17, 95% CI = 3.74–7.14), fam-trastuzumab deruxtecan (OR = 4.86, 95% CI = 3.32–7.04), idelalisib (OR = 4.74, 95% CI = 3.13–7.07) and osimertinib (OR = 3.21, 95% CI = 2.11–4.79) demonstrated a significant association with an increased risk of the outcome. These associations were highly statistically significant, with *p*-values less than 0.001.

## 4 Discussion

Eosinophilic pneumonia is an uncommon pulmonary disorder, with drug-induced eosinophilic pneumonia being even more infrequent. Presently, understanding of drug-induced eosinophilic pneumonia is primarily based on descriptive studies and case reports (14–16). This study employed data from the FAERS to identify drugs associated with an increased risk of eosinophilic pneumonia through disproportionality and multivariate analyses. A total of 15,374 cases of drug-induced eosinophilic pneumonia were identified, with no significant gender differences in incidence. The incidence rate was higher in the 45–64-year age group, with hospitalization being the most common adverse outcome. Initially, 302 drugs were screened. In the overall TTO study, drug-induced EP was found to be an early failure, with a median time to onset of 59 days (IQR: 21–149 days). Daptomycin demonstrated an earlier median onset time of 19 days, while durvalumab and idelalisib were classified as wear-out failure types. Following disproportionality analysis, univariate analysis, and LASSO regression, 56 drugs were shortlisted. Further multivariate regression analysis identified 14 drugs as associated factors for the reporting of EP. The pharmacological agents predominantly encompass immune checkpoint inhibitors, such as nivolumab, pembrolizumab, atezolizumab, and ipilimumab; targeted small molecule inhibitors, including alectinib argeting anaplastic lymphoma kinase(ALK), abemaciclib targeting cyclin-dependent kinase 4/6(CDK4/6), everolimus targeting mechanistic target of Rapamycin(mTOR), osimertinib targeting epidermal growth factor receptor(EGFR), and idelalisib targeting phosphoinositide 3-kinase delta(PI3Kδ); an antibody-drug conjugate(ADC), specifically sacituzumab govitecan; and an antiarrhythmic agent, amiodarone.

Antibiotic therapy is commonly initiated for suspected bacterial pneumonia; however, antimicrobial agents can occasionally induce adverse pulmonary reactions, manifesting as drug-associated eosinophilic pneumonia. Antimicrobial agents, notably, represent a significant proportion of published cases; however, the evidence predominantly remains anecdotal, limited to isolated case reports and small series. Among these agents, daptomycin is the most frequently and convincingly implicated (17). Daptomycin is believed to bind pulmonary surfactant, be recognized as antigen by alveolar macrophages, recruit T-helper 2 (Th2) lymphocytes, and trigger interleukin-5 (IL-5) release, thereby promoting eosinophopoiesis and their trafficking into the lungs (18, 19). In comparison to other pharmaceuticals, eosinophilic pneumonia associated with daptomycin typically manifests earlier, exhibiting a median latency period of merely 3 weeks. Our observed time-to-event data for daptomycin-induced eosinophilic pneumonia closely align with previous estimates. Upon daptomycin rechallenge, 14.9% of patients experienced relapse of eosinophilic pneumonia; notably, 57.1% of these recurrences manifested within 24 h (19). Daptomycin-associated acute eosinophilic pneumonia most commonly presents with dyspnea accompanied by eosinophilic alveolitis on bronchoalveolar lavage, followed by pulmonary infiltrates evident on chest radiography or computed tomography (20). With the increasing utilization of daptomycin, it is imperative to actively exclude eosinophilic pneumonia in all patients receiving this treatment who exhibit respiratory symptoms, regardless of age, sex, dosage, or the presence of peripheral blood eosinophilia (20).

Immune checkpoint inhibitors (ICIs) enhance antitumor immunity by inhibiting suppressive immune signals; however, they are associated with immune-related adverse events (irAEs) (21, 22). Our study has identified these agents as the primary drugs linked to drug-induced eosinophilic pneumonia. A potential mechanism for this class of drugs involves the blockade of the PD-1/PD-L2 pathway by anti-PD-1 antibodies, which alleviates the suppression of Th2 cells, facilitates the release of cytokines such as IL-5, and induces the recruitment and activation of eosinophils in pulmonary tissue. This process ultimately results in AEP as an irAE (23, 24). Moreover, drug-induced interstitial lung disease represents a diverse and heterogeneous group of conditions (25). Its characteristic radiological manifestations include various patterns such as ground-glass opacities, consolidation, and reticulonodular shadows, complicating the differentiation of drug-induced EP from other forms of drug-induced interstitial lung disease (ILD) (26). EP is diagnosed by elevated eosinophil counts in peripheral blood and bronchoalveolar lavage fluid, increased FeNO levels, and ground-glass opacities in the bilateral lower lobes on imaging, without infectious symptoms. Discontinuing the medication and starting corticosteroid therapy generally alleviates the condition, allowing for the continuation of immunotherapy with careful monitoring (27, 28). Reports of ICI-associated EP or asthma as irAEs are rare, with cases involving drugs like nivolumab, ipilimumab, durvalumab and pembrolizumab. This rarity suggests that eosinophil-associated irAEs might be under-recognized or under-reported in clinical settings (29).

The mechanisms behind drug-induced eosinophilic pneumonia vary based on the drug involved. Traditional antibiotics like daptomycin typically cause a Type IVb delayed hypersensitivity reaction, where the drug acts as a hapten, triggering a Th2 cell response and IL-5 release, leading to eosinophil activity in the lungs. This condition may also indicate a broader systemic hypersensitivity, such as DRESS syndrome. Conversely, immune checkpoint inhibitors (ICIs) like anti-PD-1/PD-L1 and anti-CTLA-4 antibodies cause eosinophilic pneumonia through a different mechanism, disrupting immune tolerance and reversing T-cell exhaustion (18, 30). ICIs, like anti-PD-1/PD-L1 and anti-CTLA-4 antibodies, fight tumors by disrupting immune tolerance and reversing T-cell exhaustion, but this can also lead to immune-related adverse events (irAEs) by impairing tolerance to self-antigens. In ICI-induced pneumonitis, eosinophil recruitment may result from a skewed Th2 inflammatory response or cross-reactivity between tumor neoantigens and normal lung antigens (31). Unlike the antigen-specific reaction seen with antibiotics, ICI-induced EP is an autoimmune-like effect marked by unregulated T-cell growth and cytokine storms in the lungs. Understanding these mechanisms is crucial for developing targeted treatments, such as using corticosteroids for ICI-EP instead of immediately stopping the drug.

Due to their advantages in efficacy and safety compared with traditional chemotherapy drugs, molecular targeting therapy have become the standard cancer treatments. To date, the number of small-molecule targeted antitumor drugs approved by the FDA and the National Medical Products Administration (NMPA) has exceeded one hundred (32, 33). Nonetheless, molecular-targeted therapies may still induce specific adverse drug reactions (ADRs) that, while infrequently severe, have the potential to be life-threatening. Consequently, the ability to predict which patients are at risk of developing ADRs following treatment with molecular-targeted therapies is becoming increasingly crucial (34). This study identified a significant correlation between targeted small molecule inhibitors—specifically alectinib, abemaciclib, everolimus, osimertinib, and idelalisib—and the incidence of eosinophilic pneumonia. This finding contrasts with earlier literature, which had generally categorized these cases under the broader term of pneumonitis. Alectinib is a second-generation ALK inhibitor approved as a first-line treatment for patients with ALK-positive non-small cell lung cancer (NSCLC). Drug-related pneumonitis (DRP), a rare but potentially severe adverse reaction, has been identified as a complication associated with its use, with an incidence rate of approximately 2.6% (35). The majority of cases manifested between 20 days and 1 year following the initiation of treatment. Upon discontinuation of the drug and administration of corticosteroids, the majority of patients exhibited improvement (36, 37). Furthermore, some cases indicated that pneumonitis did not recur even when the drug was readministered (38, 39). Idelalisib carries a black-box warning from the U.S. Food and Drug Administration (FDA) regarding the risk of pneumonitis (40). In clinical studies, severe or fatal pneumonitis occurred in 4–17% of patients treated with idelalisib, sometimes necessitating mechanical ventilation or resulting in death (40, 41). CDK4/6 inhibitors, such as abemaciclib, have drawn attention to pneumonitis as a potentially serious adverse event in Phase II and III clinical trials for the treatment of advanced or metastatic breast cancer. Data show that among patients with advanced or metastatic breast cancer treated with abemaciclib, the overall incidence of pneumonitis was 3.3%, including 0.6% of grade 3 or higher severe pneumonitis and 0.4% of fatal pneumonitis. The onset of pneumonitis varies among different CDK4/6 inhibitors, with abemaciclib exhibiting a median onset range of 50 to 250 days. The pulmonary side effects associated with mTOR-selective inhibitors, such as everolimus, are considered a class effect, indicating that they are common adverse reactions observed across all rapamycin derivatives (42). These pulmonary manifestations align with those observed with idelalisib, as they are part of the same intracellular signaling pathway involving PI3K/protein kinase B (AKT)/mTOR. A retrospective study revealed that low-grade pneumonitis, potentially or likely associated with everolimus, was relatively prevalent, occurring in 25% of the patients evaluated. Additionally, 56% of patients developed respiratory signs or symptoms related to everolimus (43, 44). In contrast to immune checkpoint inhibitors, pulmonary toxicities associated with molecular targeted therapies are generally classified as pneumonitis. This classification encompasses terms such as alveolitis, interstitial lung disease, pulmonary infiltrates, and pulmonary toxicity, and is not limited to eosinophilic pneumonia.

Over the past decade, the FDA has approved multiple ADCs for various malignancies; those associated with DRP include the solid-tumor agents fam-trastuzumab deruxtecan, sacituzumab govitecan, enfortumab vedotin, tisotumab vedotin, mirvetuximab soravtansine, and amivantamab-vmjw, as well as the hematologic agents loncastuximab tesirine-lpyl and polatuzumab vedotin-piiq (45). In our disproportionality analysis of drug induced EP, fam-trastuzumab deruxtecan was associated with 341 cases (ROR = 49.40, 95% CI: 44.26–55.13), followed by brentuximab vedotin with 71 cases (ROR = 11.20, 95% CI: 8.86–14.16), trastuzumab emtansine with 58 cases (ROR = 13.17, 95% CI: 10.16–17.07), and sacituzumab govitecan with 55 cases (ROR = 14.99, 95% CI: 11.48–19.57). The time-to-onset (TTO) study of ADC drugs revealed an early failure pattern observed across these agents, with a median time to onset ranging between 3 and 5 months. In our multivariate regression analysis, both sacituzumab govitecan (OR = 2.73, 95% CI: 1.40–4.87) and fam-trastuzumab deruxtecan (OR = 4.86, 95% CI: 3.32–7.04), were identified as factors independently associated with the reporting of EP. Reports of antibody-drug conjugate (ADC)-associated eosinophilic pneumonia (EP) are rare; however, data on drug-related pneumonitis have been documented in clinical trials across various agents. In clinical trials of trastuzumab deruxtecan, the incidence of DRP ranged from 10 to 15%, with grade 3 or higher events accounting for approximately 1% of cases (46, 47). The majority of cases occurred within 12 months. The incidence was lower with sacituzumab govitecan; in the phase III ASCENT trial, only one patient with underlying lung comorbidities, including lung metastases and radiation-induced lung fibrosis, developed grade 3 pneumonitis, with an incidence rate of only 0.4% (48). Unlike with immune checkpoint inhibitors, it is recommended to permanently discontinue ADC drugs in patients who develop grade 2 or higher pneumonitis due to the risk of progression to severe or even fatal cases (49, 50). However, further studies are needed to evaluate the safety of rechallenge in cases of low-grade drug-related pneumonitis.

In our multiple regression analysis, additional pharmacological agents identified as factors independently associated with the reporting of eosinophilic pneumonia included amiodarone. Pulmonary toxicity associated with amiodarone is a well-documented adverse reaction, with numerous pulmonary disorders reported in the literature (51). Amiodarone-induced eosinophilic pneumonia, however, is a relatively rare adverse effect, with an incidence of pulmonary toxicity from amiodarone reported to range between 5 and 13% (52). Amiodarone-associated eosinophilic pneumonia typically has a favorable prognosis. In cases where amiodarone pulmonary toxicity is suspected, patients should undergo comprehensive imaging, pulmonary function testing, and bronchoalveolar lavage. The findings from these diagnostic procedures can be instrumental in establishing a presumptive diagnosis of amiodarone-induced pulmonary toxicity (53).

In contrast to previous research, our analysis integrates contemporary methodological guidance on spontaneous reporting system-based signal detection as outlined by Duke et al., with a specific focus on addressing challenges related to disproportionality measures, data sparsity, and reporting biases (13). However, our study is subject to several additional limitations. This study recognizes that its findings are constrained by the intrinsic limitations of spontaneous reporting databases, notably notoriety bias, channeling bias, and masking effects. As a result, the high-frequency signals detected for certain drugs, such as immune checkpoint inhibitors, may represent reporting trends rather than an actual increase in risk. Consequently, our results should be interpreted as statistical associations and potential safety signals that necessitate further validation, rather than as established causal relationships. A major limitation is the challenge in defining and validating eosinophilic pneumonia (EP) in the FAERS database, as its diagnosis relies on clinical symptoms, specific radiological patterns, and occasionally bronchoalveolar lavage (BAL) results, which are not accessible in spontaneous reports. In our effort to identify as many reliable cases of drug-related EP as possible, we conducted disproportionality analyses alongside univariable and multivariable screenings of the FAERS database. Given the extreme rarity of drug-induced EP, the signals we identified cannot be considered comprehensive. Reporters often categorize pulmonary toxicity under preferred terms such as “interstitial lung disease” or “acute respiratory distress syndrome,” which may result in missed or miscoded cases. Similar to the case with immune-checkpoint inhibitors, where pulmonary adverse events are generally labeled as “pneumonitis” rather than the specific EP we targeted, some degree of misclassification is unavoidable. Although a subset of published case reports excluded alternative causes of eosinophilic pneumonia, not all underwent thorough causality assessment. Furthermore, as is inherent to spontaneous reporting systems, there was a substantial proportion of missing or incomplete temporal data (84.7%), which restricted our time-to-onset (TTO) analysis to a smaller subset of the overall cohort. While this limits the generalizability of the TTO findings, retaining the full cohort for the primary disproportionality analysis ensured the robustness of the signal detection. Lastly, despite employing scientifically robust methods to address missing or anomalous data fields, residual incompleteness in the database may have influenced our findings.

## 5 Conclusion

As the understanding of drug-induced eosinophilic pneumonia (EP) deepens, the number of reported cases continues to rise. The respiratory symptoms of EP can rapidly progress from mild manifestations to severe, life-threatening respiratory failure. Clinicians should be highly vigilant for EP in patients taking medications with the potential to induce this condition, particularly when new pulmonary infiltrates are detected along with peripheral or bronchoalveolar eosinophilia. An accurate diagnosis necessitates a comprehensive assessment of the timing of drug exposure, imaging results, bronchoalveolar lavage (BAL) eosinophils, and the exclusion of other possible causes. Our study offers substantial data on drugs associated with eosinophilic pneumonia, facilitating earlier identification and better management of EP. Future research should concentrate on clarifying the immune mechanisms underlying drug-related EP, while ongoing clinical trials and real world data should be employed to improve risk assessment and monitoring.

## Data Availability

The datasets presented in this article are not readily available because our data is sourced from a public database and is accessible. Requests to access the datasets should be directed to FDA Adverse Event Reporting System and Vigibase.
